# CASSIA (cardiology software suite for image analysis): a potential new tool for the evaluation of [^18^F]FDG PET/CT in the setting of infective endocarditis

**DOI:** 10.1007/s11548-022-02729-6

**Published:** 2022-09-02

**Authors:** David Palomino-Fernández, Adolfo Gómez-Grande, Mirene Fernández-Igarza, Patrick Pilkington, Alexander P. Seiffert, Héctor Bueno, Enrique J. Gómez, Patricia Sánchez-González

**Affiliations:** 1 Biomedical Engineering and Telemedicine Centre, ETSI Telecomunicación, Center for Biomedical Technology, Universidad Politécnica de Madrid, Madrid, Spain; 2 Department of Nuclear Medicine, Hospital Universitario 12 de Octubre, Madrid, Spain; 3 Facultad de Medicina, Universidad Complutense de Madrid, Madrid, Spain; 4 Cardiology Department and Instituto de Investigación Sanitaria (imas12), Hospital Universitario 12 de Octubre, Madrid, Spain; 5 Centro Nacional de Investigaciones Cardiovasculares (CNIC), Madrid, Spain; 6 Centro de Investigación Biomédica en Red de Enfermedades Cardiovasculares (CIBERCV), Madrid, Spain; 7 Centro de Investigación Biomédica en Red de Bioingeniería, Biomateriales y Nanomedicina (CIBER-BBN), Zaragoza, Spain

**Keywords:** Infective endocarditis, Prosthetic valve endocarditis, [^18^F]FDG PET/CT, Myocardial metabolism quantification, Computer-aided diagnosis

## Abstract

**Purpose:**

Due to the high morbidity and mortality of infective endocarditis (IE), medical imaging techniques are combined to ensure a correct diagnosis. [^18^F]FDG PET/CT has demonstrated the ability to improve diagnostic accuracy compared with the conventional modified Duke criteria in patients with suspected IE, especially those with prosthetic valve infective endocarditis (PVIE). The aim of this study is to provide an adjunctive diagnostic tool to improve the diagnostic accuracy in cardiovascular infections, specifically PVIE.

**Methods:**

A segmentation tool to extract quantitative measures of [^18^F]FDG PET/CT image studies of prosthetic heart valve regions was developed and validated in 20 cases of suspected PVIE, of which 9 were confirmed. For that, Valvular Heterogeneity Index (VHI) and Ring-to-Center Ratio (RCR) were defined.

**Results:**

Results show an overall increase in the metabolic uptake of the prosthetic valve ring in the studies with confirmed PVIE diagnosis (SUV_max_ from 1.70 to 3.20; SUV_mean_ from 0.86 to 1.50). The VHI and RCR showed areas under the curve of 0.727 and 0.808 in the receiver operating characteristics curve analyses, respectively, for PVIE diagnosis. Mann–Whitney U tests showed statistically significant differences between groups for RCR (p = 0.02). Visual analyses and clinical reports were concordant with the extracted quantitative metrics.

**Conclusion:**

The proposed new method and presented software solution (CASSIA) provide the capability to assess quantitatively myocardial metabolism along the prosthetic valve region in routine [^18^F]FDG PET/CT scans for evaluating heart valve infectious processes. VHI and RCR are proposed as new potential adjunctive measures for PVIE diagnosis.

**Supplementary Information:**

The online version contains supplementary material available at 10.1007/s11548-022-02729-6.

## Introduction

Infective endocarditis (IE) is an inflammatory and proliferative disease affecting the heart valves. IE results from infection, usually bacterial, of the endocardial surface of the heart, entailing a high risk of mortality and morbidity [1]. Currently, modified Duke criteria are considered as the gold standard in the diagnosis of IE. These criteria provide a diagnostic outcome classified in *definite*, *possible*, or *rejected* IE based on clinical, echocardiographic, microbiological and pathological findings [2, 3]. However, the sensitivity of these criteria is compromised (approximately 80%) by the difficulty of the echocardiography interpretation and the identification of the microorganisms [4–6]. Indeed, blood cultures turn out to be negative in 5–70% of IE cases, classifying these patients as *possible IE* [6–8]. Transthoracic echocardiography (TTE) and transesophageal echocardiography (TEE) are recommended as part of the evaluation of IE. TTE shows an overall sensitivity of 70% for the diagnosis of native valve endocarditis (NVE) decreasing this percentage to 50% in prosthetic valve endocarditis (PVIE). Sensitivity reaches higher values when TEE is performed for the diagnosis of NVE and PVIE (96 and 92%, respectively) [4]. However, echocardiography sensitivity can be variable depending on the expertise of the clinician, vegetation size, stage of the disease, or even the prosthetic material. Moreover, changes may not be apparent until there is structural damage from the infectious process [4, 9].

[^18^F]FDG PET/CT has shown promise as one of the tools in the evaluation of cardiovascular infections [9–15], particularly in patients with suspected prosthetic valve infective endocarditis (PVIE) [2, 16–19]. However various clinical factors such as the type of microorganism causing the infection, small vegetation size, prior antimicrobial therapy or non-standardization of acquisition and reconstruction protocols may have a negative impact on [^18^F]FDG PET/CT results. Additionally, adequate patient preparation and suppression of physiologic myocardial activity through low carbohydrate and high fat diet protocols [20] must be followed strictly to achieve reliable results [9–11, 16]. Nevertheless, recent studies have proved that the addition of [^18^F]FDG PET/CT as a major criterion in the modified Duke Criteria has the potential to increase sensitivity of the diagnosis by reclassifying *possible IE* cases as *definite* or *rejected IE* [16, 21]. [^18^F]FDG PET/CT images are visually evaluated in search of abnormal uptake and intensity patterns of increased activity at the site of infection. In addition to qualitative analysis, semi-quantitative analysis can also be performed. The standardized uptake value (SUV) and other parameters (semi-quantitative ratios) have been used to attempt to optimize the diagnosis of cardiovascular infections [2, 21, 22]. While SUV metrics have been extensively validated in oncologic applications of [^18^F]FDG PET/CT, there is currently no standardized methodology for obtaining SUV and other semi-quantitative parameters for cardiovascular infections and the reported values are not yet consistent between studies [9, 23].

In this study, the Cardiological Software Suite for Image Analysis (CASSIA) is proposed for the qualitative and quantitative analysis of prosthetic valve uptake in [^18^F]FDG PET/CT scans acquired during clinical monitoring of patients with suspected PVIE. Quantitative parameters that describe the myocardial metabolism in the prosthetic ring regions are defined. A technical validation is performed using [^18^F]FDG PET/CT image studies of patients meeting the age and sex standards in which PVIE is most likely to develop.

## Materials and methods

### Image studies

Retrospective analysis of [^18^F]FDG PET/CT images provided by the Nuclear Medicine Service of the Hospital Universitario 12 de Octubre, Madrid, between 2019 and 2021 was performed. A total of 20 image studies are available to perform the technical validation. Among the 20 studies, there are 13 males and only 3 females. This accounts for a total of 16 patients (13 single scan, 2 double scans, 1 triple scan). The mean age was 77.45 ± 11.37. Therefore, the tool is validated in a sample of 20 image studies meeting the age and sex standards in which PVIE is most likely to develop [24]. Among the 20 studies, 9 of them have confirmed PVIE.

### Image acquisition

All [^18^F]FDG PET/CT scans have been acquired between 2019 and 2021. This change in protocol means that all patients have followed a strict diet low in carbohydrates and high in fat to optimize FDG uptake. All scans were performed using a SIEMENS Biograph 6 True Point [^18^F]FDG PET/CT scanner (Siemens Healthineers, Erlangen, Germany). The initial position of the patient relative to the imaging equipment when acquiring the images was HFS (Headfirst Supine) and in all cases, whole body images were obtained. A mean dose of 308.23 ± 91.69 MBq of [^18^F]FDG was injected, and the images were corrected for attenuation with CT. Images were reconstructed with a point spread function (PSF) algorithm (3 iterations, 21 subsets, all-pass filter), and scatter and random correction were performed. Reconstructed PET image matrix size is 168 × 168 pixels, with 4.0728 mm pixel spacing and 16 bits per pixel (bpp). The CT images have a resolution of 512 × 512 pixels with 0.9766 mm pixel pitch and 12 bpp.

### Image processing

An image processing methodology that begins with the co-registration and pre-processing of PET and CT images is defined. PET images are therefore interpolated to obtain the same resolution as in the anatomical images. The SPM12 (Statistical Parametric Mapping) software is used to co-register the CT and PET images [25]. While for the technical validation, the images are co-registered as described above, it has since been included in the tool as an automated step once the image files are loaded [26]. Once the images have been co-registered, they need to be pre-processed.

First, a conversion of the gray intensity values of the PET image expressed in activity concentration to SUV values is performed, for convenience in subsequent calculations and parameter derivation. Then, a subvolume is generated so that approximately, the lower half and the upper fifth of the whole-body CT image are cut off, aiming a more precise visualization and segmentation of the heart. The final volumes must be interpolated so that the original dimensions are ensured during visualization and quantification. The fusion images combining CT and PET studies are then generated so it is possible to assess simultaneously [^18^F]FDG uptake and anatomical information.

**Fig. 1:**
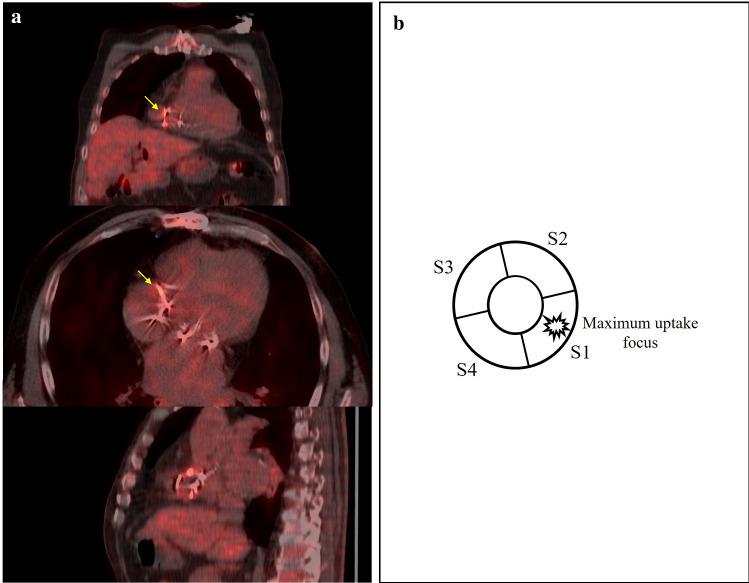
** a** [^18^F]FDG PET/CT image in coronal, axial and sagittal slices showing focal enhancement over the aortic prosthetic valve, difficult to objectify and localize in the visual analysis. **b** Prosthetic ring correct orientation and definition of the 90° segments. Counterclockwise definition of the 90° segments regarding to the maximum uptake focus, allocated in the center of the first segment

A subvolume containing the prosthetic valve is segmented to adequately visualize the valvular region and to exclude close malignant or suspicious pathological masses or nodules that might interfere with the analysis. Once the subvolume is generated, the orientation of the image plane is necessary to precisely visualize the valvular plane. The correct orientation is achieved when the coronal plane’s normal vector is aligned with the valve’s front-face normal vector, while the axial and sagittal image planes show the top and lateral views of the valve. Nevertheless, the orientation process may be heterogeneous between studies due to anatomical disparities of the patients (Fig. 1). Therefore, volume rotation may be required to ensure the correct visualization of the valvular plane. In preference to minimize the derived interpolation artifacts from rotation operations and to preserve original image features, a unique rotation is applied. By means of Euler’s theorem, the equivalent rotation angle and axis are calculated as from the history of rotations conducted by the user. This proceeding is applied on every step of the orientation process, so as the correction of the interpolation artifact is displayed in real time.

Once the definite valvular plane is defined, a three-dimensional toroid-shaped mask is generated to segment the metabolic activity of the prosthetic ring. Then, the quantitative parameters of metabolic activity, SUV_max_ and SUV_mean_, are extracted from both inner and outer sections of the toroid. Additionally, these quantitative metrics are computed of four 90º segments defining anterior, posterior, and lateral portions of the segmented valve. As mentioned, anatomical disparities between patients may result in different valve plane orientations so that the anterior, posterior, and lateral portions definition is not strictly adhered. However, to advantageously locate the anatomical reference points with respect the prosthetic valve, a maximum intensity projection image of the oriented CT volume is provided. Moreover, to increase the reproducibility and interpretation of the results, a criterion is followed, allocating the maximum uptake focus inside the first defined segment (Fig. 1). This means that the maximum uptake focus does not fall right in the space between two segments. In this manner, the focal nature of the uptake is preserved, adequately reflecting the heterogeneity between segments. The rest of the segments are then calculated regarding the first one, counterclockwise.

Finally, this study proposes two ratios based on the recognition of heterogeneity uptake patterns along the prosthetic ring as potential diagnostic biomarkers of IE. The Valvular Heterogeneity Index (VHI) is defined as the maximum uptake value (SUV_max_ of the first segment S1, where the focus of maximum uptake is positioned) divided by the mean uptake of the remaining segments (S2-S4), as shown in (1). The Ring-to-Center Ratio (RCR) is defined as the maximum uptake of the prosthetic ring divided by the maximum uptake of the central portion of the valve as a reference uptake point, as shown in (2).

1 $$Valvular Heterogeneity Index \left(VHI\right)=\frac{{SUV}_{\mathit{max}S1} }{mean({SUV}_{mean S2-S4})}$$

2 $$Ring-to-Center Ratio (RCR)=\frac{{SUV}_{\mathit{max}RING}}{{SUV}_{\mathit{max}CENTER}}$$

### Statistical analysis

Quantitative variables are represented as mean ± standard deviation. Statistical analysis was performed separating the patients according to the diagnosis confirmation of IE. Differences of SUV_max_, SUV_mean_, and valvular heterogeneity ratios (RCR and VHI) between groups were studied with Mann–Whitney U test. *P* values < 0.05 were considered statistically significant, and statistical analyses were performed in SPSS software version 19.00 (IBM Corp., Armonk, NY).

## Results

### CASSIA: [^18^F]FDG PET/CT processing tool

The proposed image-processing algorithm is implemented in MATLAB R2020b. A graphical user interface is developed based on MATLAB’s App Designer environment. Individual modules are dedicated to each distinct sections described within the methodology. Additionally, the SPM12 (Statistical Parametric Mapping) software is used to co-register the CT and PET images [25]. While for the technical validation, the images are co-registered as described above, it has since been included in the tool as an automated step once the image files are loaded [26].

**Fig. 2:**
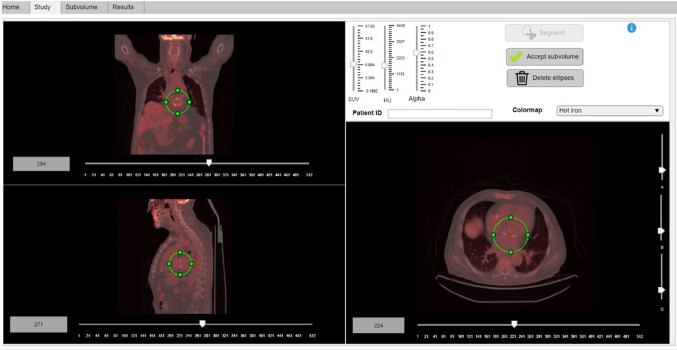
*Study module*. The user is expected to define an ellipsoidal subvolume encompassing the heart valve area of interest using the three interactive ellipses

Initially, the algorithm imports the co-registered volumes. Then, three axes are enabled for the visualization of the axial, sagittal and coronal fusion images. The segmentation of a subvolume containing the prosthetic ring, excluding close masses or nodules, is performed in this module (Fig. 2). Each of the axial, sagittal and coronal axes have an associated slider bar below to navigate along the volume slices. On the other hand, the vertical sliders modify the display window of the PET and CT images. Additionally, an alpha value is modifiable to alter the transparency property and alternate between PET and CT images. Different colormaps for the visualization of [^18^F]FDG PET/CT images are eligible.

The *Segment* button generates a two-dimensional ellipse in each of the visualization planes. The vertical sliders at the right hand of the axial plane are aimed to modify the different radiuses to refine the valvular segmentation. The movement and resizing of the three ellipses occur in a solid manner. This coordinated movement of the three ellipses within the three-dimensional [^18^F]FDG PET/CT image space enables to form a valid ellipsoidal subvolume encompassing the prosthetic valve. Once the ellipses have been adequately positioned, by pressing the *Accept subvolume* button, the segmentation of the defined ellipsoidal subvolume is performed.

**Fig. 3:**
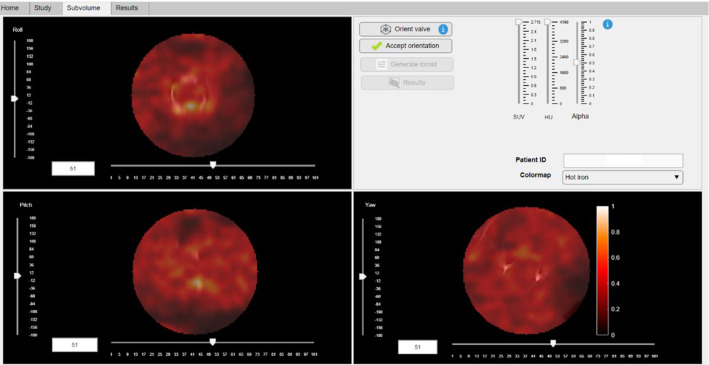
*Subvolume module*. The user is expected to define the adequate orientation of the prosthetic valve. For this purpose, vertical sliders 2D-approach and the *Orient Valve* button 3D-approach permit two different and equivalent orientation methodologies

**Fig. 4:**
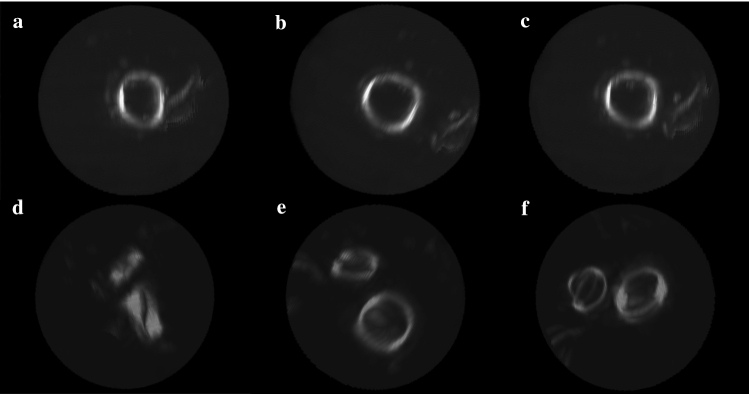
3D-visualization window for the precise orientation of the prosthetic valve. The user is expected to manually click and drag the volume and achieve the desired position of the valve. Images **a**, **b** and **c** show the orientation process view, ending with the desired front-faced view of the valve. Images **d**, **e** and **f** show the orientation process with a 2-valve (aortic and mitral) study. Image e shows the adequate orientation of the mitral valve, whereas image f shows the correct orientation of the aortic valve

Subsequently, [^18^F]FDG PET/CT images in the axial, sagittal and coronal planes of the segmented ellipsoidal subvolume are displayed. The *Subvolume module* (Fig. 3) aims the precise orientation of the valve to perform a robust segmentation of the prosthetic valve. For this purpose, the vertical sliders are used to rotate the subvolume with a two-dimensional visualization approach on each axis. As mentioned, the real-time interpolation artifact correction is applied every time the user interacts with the vertical rotation sliders. Additionally, the *Orient valve* button enables the user to orient the subvolume in a 3D interactive visualization window, showing the maximum intensity projection (MIP) of the generated CT subvolume (Fig. 4). The user clicks and drags the valve subvolume to achieve the adequate orientation, facing the valve to the visualization plane. Once more, a unique rotation along the precise axis is applied to minimize interpolation artifacts.

**Fig. 5:**
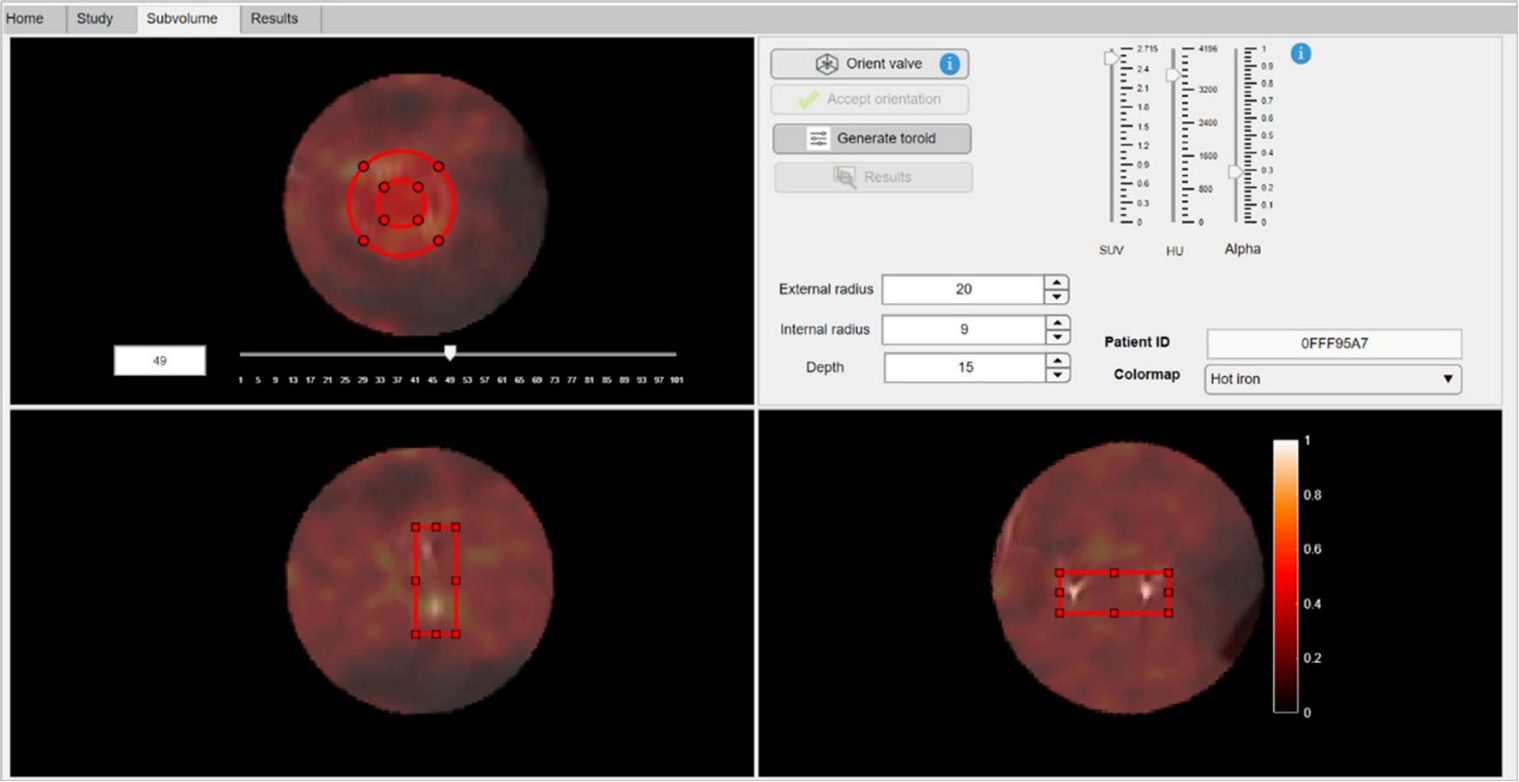
*Subvolume module*. Once the adequate orientation of the valve is achieved, the two-dimensional structures that make up the three-dimensional toroid are displayed. The user is expected to precisely locate the toroidal mask encompassing the prosthetic valve, being able to customize the size properties of the 3D mask

The *Accept Orientation* button automatically generates the two-dimensional structures that conform the three-dimensional toroid segmentation mask (Fig. 5). A predefined toroid is provided regarding to the size of the user-defined subvolume. However, the toroid may be repositioned and reshaped to fit to the extension of the prosthetic ring and perform a refined segmentation of the valve. To this end, the tool features three spinners to specify the inner radius, outer radius, and depth of the three-dimensional toroid. It is worth mentioning that the slice displayed in the coronal axes is the top face of the toroid, not necessarily corresponding to the maximum uptake plane within the 3D mask.

**Fig. 6:**
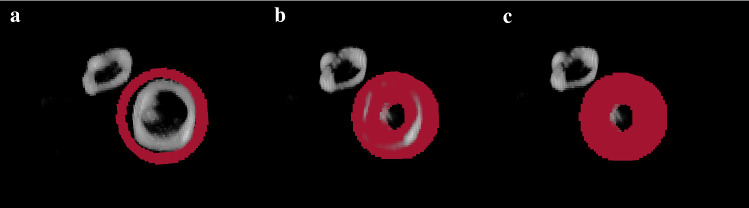
3D-visualization window to verify the adequation of the defined mask to the prosthetic ring obtained by means of the CT-based MIP subvolume. The user is expected to manually click and drag the volume to check for un-segmented valve regions. Moreover, the customization of the mask outer and inner radiuses (shown in **a** and **b**) and depth (shown in **c**) allows further refining the segmentation of the prosthetic ring

Once the toroid is correctly centered to the prosthetic ring, the *Generate toroid* button displays a 3D visualization window of the CT MIP image of the valve and the toroidal defined mask. An additional slider is supplied to modify the display threshold of the MIP image, so that the finest visualization of the valve is ensured. This methodological step aims to provide the user a pre-visualization of the generated mask which the quantitative image features are extracted from. As shown in Fig. 6, the module follows the same click and drag approach allowing the user to detect possible portions of the valve getting out of the defined mask. The toroid size may be then modified to tightly embrace the valve for robust segmentation and quantification results.

**Fig. 7:**
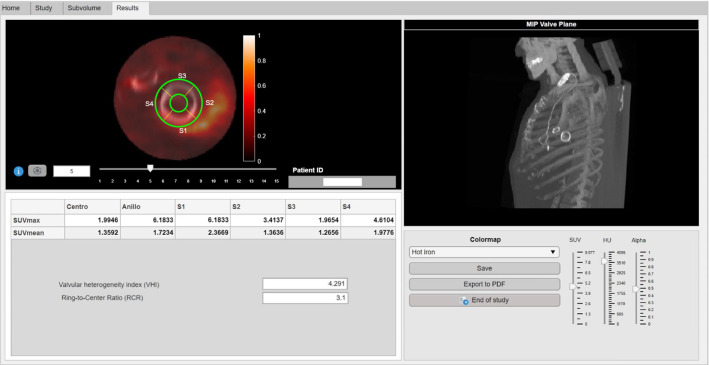
*Results module*. The exploration of the performed segmentation to adequately interpret the obtained results is aimed at this module. The fusion image of the subvolume encompassing the heart valve is shown. To anatomically localize the valve-segments, a maximum intensity projection image of the valve plane is shown. Quantitative SUV image features regarding to the segmentation regions as well as heterogeneity defined ratios are provided. Data exporting options are also permitted

When pressing the *Results button,* the tool automatically segments the defined toroid volume from the SUV images and computes the corresponding quantitative metrics. SUV_max_ and SUV_mean_ measures of the inner and outer regions of the toroid are computed. Furthermore, the extracted quantitative metrics are computed for the 90° segments in the anterior, posterior, and lateral portions of the valve (regarding specific patient orientation). The segments are computed so that the maximum uptake focus is always positioned in the center of the first segment. The visualization of the final segmentation within the toroid pre-defined mask encompassing the prosthetic valve is performed (Fig. 7). A table with the quantitative measures extracted within the pre-defined regions is provided.

### Validation results

**Fig. 8:**
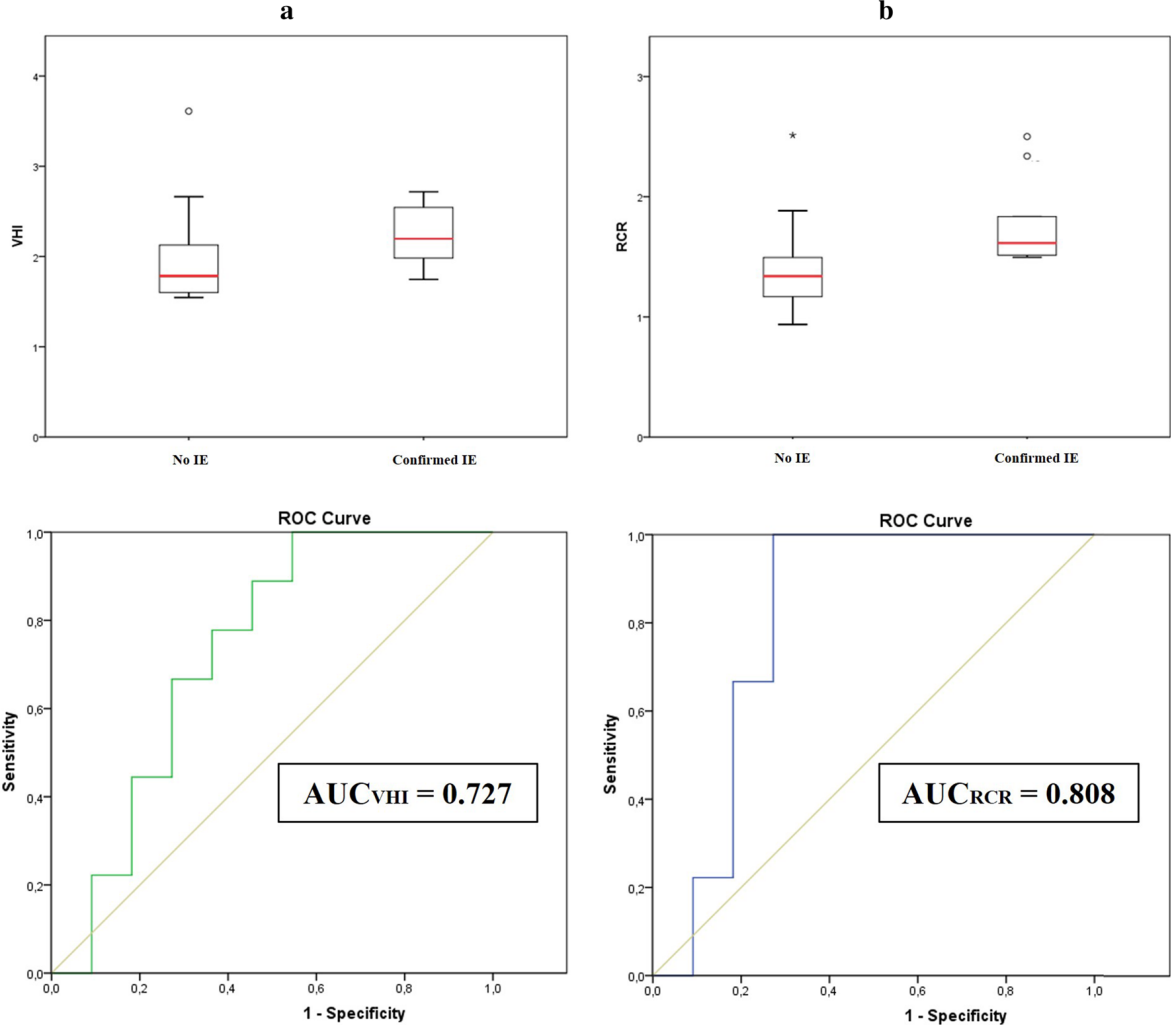
Boxplots and ROC curves for VHI **a** and RCR **b**

The patients were separated in two groups according to the diagnosis confirmation of IE. Table 1 shows the mean and standard deviation of the uptake metrics in the main valvular regions for the different groups. An overall increase in the myocardial metabolic uptake is observed in the confirmed IE group (Table 1). As shown in Fig. 8, increased values of VHI and RCR can be also noticed within the confirmed IE diagnosis group. Mann–Whitney U test is performed to verify these patterns (Table 1). Despite the observed increased uptake heterogeneity (higher VHI and RCR) tendency for the confirmed IE studies, statistically significant differences are only observed between groups for RCR.

**Table 1:** Descriptive statistics for the separated groups along the main valvular regions. Statistics for the uptake metrics and ratios are calculated. Statistically significant *p* value coefficients are given in bold

Metric	No IE	Confirmed IE	*p* value (Mann–Whitney)
SUV_max_ center	1.26 ± 1.09	1.96 ± 1.52	0.47
SUV_max_ ring	1.70 ± 1.54	3.20 ± 2.45	0.21
SUV_mean_ center	0.80 ± 0.70	1.16 ± 0.84	0.34
SUV_mean_ ring	0.86 ± 0.70	1.50 ± 1.08	0.24
VHI	2.03 ± 0.63	2.24 ± 0.35	0.09
RCR	**1.43 ± 0.44**	**1.79 ± 0.38**	**0.02**

Differences in valve heterogeneity ratios (VHI, RCR) between groups were studied with the receiver operating characteristic (ROC) analysis. Figure 8 shows the ROC curves for VHI and RCR. Both indices present an AUC above 0.7 (AUC_VHI_ = 0.727; AUC_RCR_ = 0.808).

## Discussion

Evidence is growing for [^18^F]FDG PET/CT as an adjunctive tool for the quantification of the myocardial metabolic activity related to cardiovascular infections [9–11]. Specifically, some studies have demonstrated its usefulness in the assessment of patients with suspected PVIE [2, 16, 17]. The addition of [^18^F]FDG PET/CT as a major criterion in the modified Duke Criteria has been proved to increase the diagnostic accuracy, specifically in the *possible IE* patients [16, 21]. In this study, an image processing tool to obtain quantitative and semi-quantitative metrics [2, 21, 22] based on prosthetic valve uptake in [^18^F]FDG PET/CT scans acquired during clinical monitoring of patients with suspected PVIE has been evaluated.

The quantitative metrics showed an increased myocardial metabolic uptake along the different valve regions in the confirmed IE group, which is in the line with the findings of other studies [9, 21]. Additionally, the proposed heterogeneity metrics VHI and RCR showed higher values for the positive IE group, indicating the presence of a high-uptake focus. It should be mentioned that three outliers out of twenty case studies are observed, which is not negligible. However, this could be explained by the limited size and retrospective nature of the study cohort. Nevertheless, the two case outliers with respect to the *Confirmed IE* group demonstrate that higher RCR values are related to IE confirmation, as expected. The case outlier with respect to the *No IE* group showed myocardial uptake in the left ventricle, masking valve activity and increasing the ratios. While statistically significant differences could be only observed between groups for RCR, ROC analyses revealed AUCs above 0.7 for both heterogeneity ratios (AUC_RCR_ = 0.808; AUC_VHI_ = 0.727). Further analyses were performed to include the descending aorta blood pool ratio described in the literature [2, 11, 22, 27]. However, the results seem to not improve the diagnostic accuracy of the RCR for our study population (see Supplementary Material). It should be stated that VHI and RCR are aimed at identifying heterogeneous uptake patterns throughout the valvular annulus, that could add value to the quantitative assessment of the infectious process. Tanis et al. [2] found that SUV_max_ was significantly higher in patients with *definite* PVIE in comparison with *possible* or *rejected* PVIE. However, the prosthetic valve-to-background SUV_max_ ratio (SUV ratio = SUV_max_/SUV_max_ atrial blood-pool) did not show statistically significant differences between groups. Granados et al. [11] studied the diagnostic accuracy of [^18^F]FDG PET/CT in patients with suspected IE and Implantable Cardiac Electronic Device (ICED) infection. SUV_max_ and SUV ratios (SUV ratio 1 = SUV_max_/blood-pool SUV_mean_; SUV ratio 2 = SUV_max_/liver SUV_mean_) demonstrated an overall sensitivity of 91% and specificity of 94%. Pizzi et al. [22] evaluated the SUV_max_ and prosthetic material-to-background SUV ratio (SUV ratio = SUV_max_/SUV_mean_ aortic blood) through ROC curves. A sensitivity of 91% and specificity of 79% were obtained for the SUV_max_ metric, while the SUV ratio demonstrated an overall sensitivity of 91% and specificity of 76%, with an AUC of 0.89. Swart et. al [27] obtained the ROCs for SUVmax and SUV ratio (SUV ratio = SUVmax/SUVmean aortic blood) in scans of patients suspected of PVE. As a semiquantitative measure of FDG uptake, SUV ratio of ≥ 2.1 was a 75% sensitive and 86% specific predictor of PVE, showing an AUC of 0.83. All the above indicates that the parameters studied might be useful to increase the diagnostic sensitivity of PVIE. In future works, additional quantitative features will be extracted, with special emphasis on radiomic features to obtain potential risk predictors of PVIE.

**Fig. 9:**
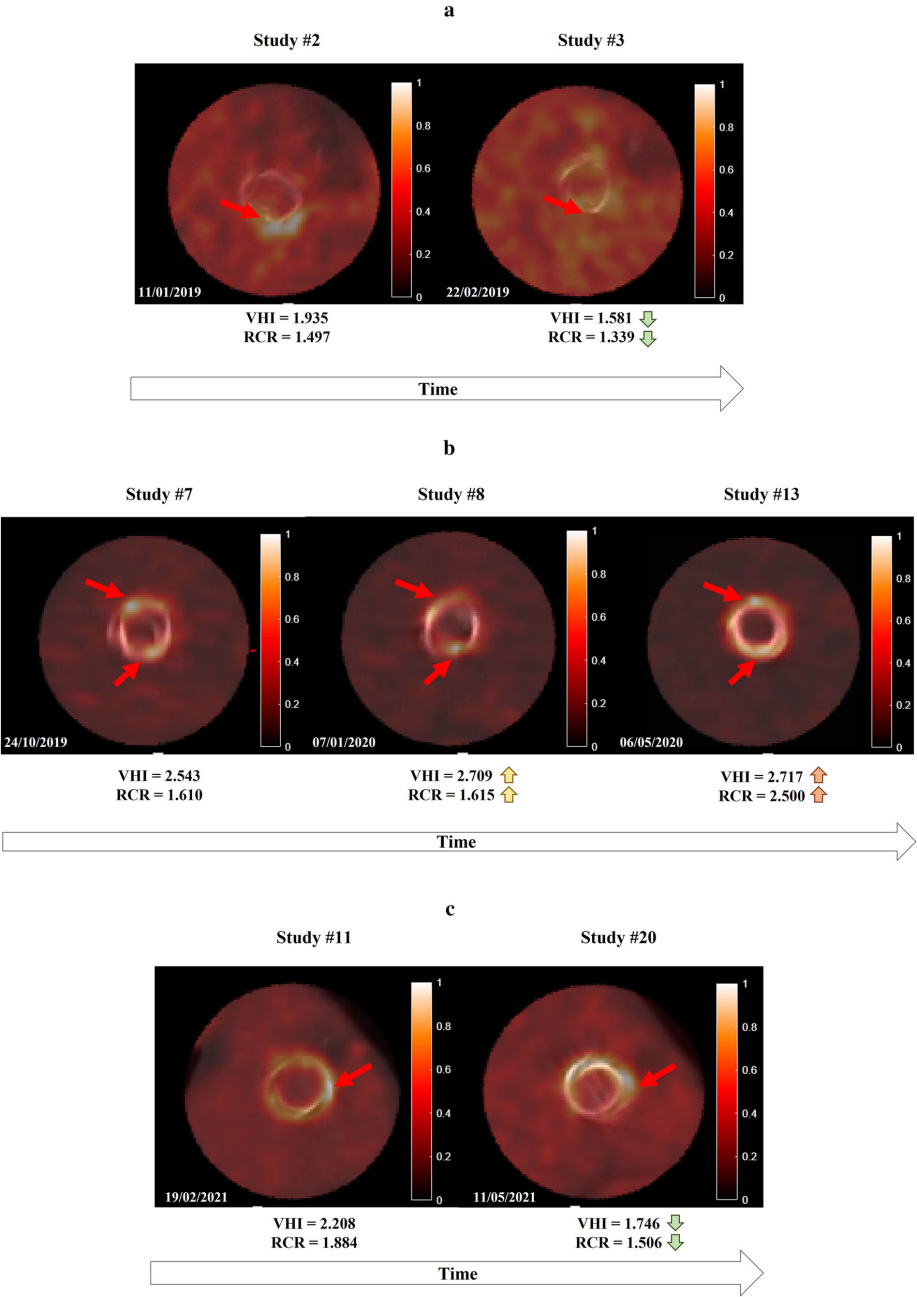
Heterogeneity ratios VHI and RCR progression. **a** The confirmed IE is resolved from the first study to the second one. Visual analysis demonstrates how the focus of maximum uptake disappears. In the same manner, VHI and RCR decrease. **b** Confirmed IE persists along the three different image studies. Visual analysis suggests an increase in uptake along the complete valvular ring, with no resolution of the infective process. VHI and RCR also follow an increasing tendency. **c** The IE diagnosis is confirmed in the second study of the series. Visual analysis suggests an increased uptake focus on the lateral valvular region. However, VHI and RCR experiment a decreasing tendency, suggesting a possible improvement of the infective process

The developed image processing tool and proposed quantitative metrics may also be used to evaluate progression of the infectious process and changes in the valvular metabolic activity. In three cases, two [^18^F]FDG PET/CT scans of the same patient from different time points are available in three cases (see Fig. 9). Heterogeneity uptake ratios were contrasted against visual analysis and the diagnosis described in the clinical reports. In the first case, clinical and metabolic resolution of PVIE process occurs. Visual analysis is in accordance with the decrease in VHI and RCR, as the main uptake focus disappears. The second case develops metabolic uptake persistence. Visual analysis suggests an increase in uptake along the complete valvular ring, with no resolution of the infective process. This patient was under antibiotic therapy cycles. The clinical report describes a *possible IE* case resolution, suggesting potential inflammatory valve activity. VHI and RCR also follow an increasing tendency. As stated in the literature [28–30], uptake with homogeneous distribution throughout the valve is interpreted as inflammatory, due to a physiological phenomenon of foreign body reaction (macrophages reacting to prosthetic material without infection and without damage to the tissue). Conversely, irregular uptake with foci of increased metabolic activity in one or more areas of the periprosthetic tissue is interpreted as a focus of infection. Finally, the third case is described as *confirmed IE* in the second study of the series. However, the clinical report suggests probable metabolic uptake affectation due to the severe condition. No significant uptake changes are observed within the valvular region, as described in the clinical report, which also suggests a non-infectious cause. VHI and RCR experiment a decreasing tendency, in line with the clinical observations. This finding indicates an adequate re-classification of a *possible IE* as *rejected IE*, based on the metabolic activity changes [22]. All the above seems to indicate agreement between visual analyses and quantitative measurements describing metabolic changes due to the infectious processes.

As mentioned in previous sections, the orientation process may be heterogeneous between studies due to anatomical disparities of the patients. Additionally, this may result in different orientations of the valvular plane so that the anterior, posterior, and lateral portions definition is not strictly adhered. Henceforth, a fully automated workflow would be preferred. The orientation of the images is frequently time-consuming and a source of variability process. The proposed image processing tool is aimed as a semi-automatic straightforward reproducible methodology to facilitate the quantification of the valvular uptake, specifically in the challenging cases. An anatomical reference such as the aortic lumen or sternum could be used to further automate the orientation process in future works, being true to the definition of the valve segments.

On the other side, the limitations of the study are mainly related to the retrospective nature of the study group and the not numerous study cohort, which could explain the lack of further statistically significant results. While successfully performing the technical validation of the developed tool, further studies need to be carried out to validate the usefulness of the quantitative metrics, especially the VHI and RCR. The presence of isolated uptake foci along various segments, or high uptake “bleed” into the inner region of the valve would compromise the robustness of the proposed indices. Further studies will be required, so the proposed heterogeneity indices could be complemented by texture analysis along the annulus, with the objective of understanding the uptake distribution and identifying potential diagnostic markers based on uptake heterogeneity. Additionally, some variables such as the diet or treatment strategies could not be controlled in this retrospective study group, and which are known to affect the myocardial glucose uptake [20]. Image studies with inadequate suppression of the myocardial uptake may result in a lack of robustness in the quantification of the valvular activity, especially in the close-to-heart regions. Further standardization in patient preparation protocols, image acquisition, reconstruction, and analysis, is required to establish semi-quantitative parameters as a helpful adjunctive tool in the diagnosis of IE. Therefore, prospective studies are necessary to validate the preclinical findings of the usefulness of [^18^F]FDG PET/CT for the diagnosis of PVIE. Future studies will focus on the validation of the proposed tool in a prospective cohort.

## Conclusions

Complications in the detection of PVIE often cause a crucial delay in the diagnosis and treatment of the disease, which may result in severe condition of the patient. The inclusion of [^18^F]FDG PET/CT as a major criterion in the modified Duke Criteria, based on visual interpretation, may have a definite impact on the diagnosis sensitivity of IE. The possibility of assessing quantitatively the prosthetic valve activity in scheduled protocol [^18^F]FDG PET/CT scans is an engaging opportunity for the detection of PVIE with important clinical implications. An image processing tool (CASSIA) for the qualitative and quantitative analysis of prosthetic valve metabolism from clinical routine [^18^F]FDG PET/CT scans based on existing standards is presented. The proposed heterogeneity uptake ratios VHI and RCR may be a new complementary measure to diagnose PVIE.

## Availability of data and material

The datasets used and/or analyzed during the current study are available from the corresponding author on reasonable request. The code is available from the corresponding author on reasonable request.

## Supplementary Information

Below is the link to the electronic supplementary material.

Supplementary file (DOCX 87 kb)
