# Supplementary material for: CASSIA (cardiology software suite for image analysis): a potential new tool for the evaluation of [18F]FDG PET/CT in the setting of infective endocarditis
Source: Int J Comput Assist Radiol Surg. 2022 Sep 2;18(1):157–69. doi: 10.1007/s11548-022-02729-6 (PMC9883360; doi:10.1007/s11548-022-02729-6)
Supplement: Supplementary file 1 — Supplementary file (DOCX 87 kb) [file 11548_2022_2729_MOESM1_ESM.docx]

# **Supplementary material**

**Table S1.** Quantitative metrics of myocardial uptake along the different valve regions. Valvular heterogeneity ratios are computed. Metrics for studies with confirmed IE diagnosis are given in bold.

| #Study | SUVmax | | | | | |  | | SUVmean | | | | | | Ratios | |  | IE diagnosis |
| --- | --- | --- | --- | --- | --- | --- | --- | --- | --- | --- | --- | --- | --- | --- | --- | --- | --- | --- |
|  | Centre | Ring | S1 | S2 | S3 | S4 | Centre | Ring | | BP | S1 | S2 | S3 | S4 | RCR | VHI | BPR |  |
| #1 | 2,26 | 2,51 | 2,51 | 2,10 | 2,43 | 2,06 | 1,33 | 1,46 | | 1,94 | 1,56 | 1,37 | 1,47 | 1,39 | 1,11 | 1,78 | 1,296 | - |
| #2 | **1,94** | **2,90** | **2,90** | **2,79** | **1,90** | **2,25** | **1,46** | **1,61** | | **1,31** | **2,00** | **1,54** | **1,38** | **1,58** | **1,50** | **1,94** | **2,215** | **Confirmed** |
| #3 | 1,91 | 2,56 | 2,56 | 2,13 | 2,14 | 2,16 | 1,52 | 1,68 | | 1,41 | 1,84 | 1,64 | 1,63 | 1,58 | 1,34 | 1,58 | 1,815 | - |
| #4 | 1,99 | 5,01 | 5,52 | 3,25 | 1,77 | 4,31 | 1,43 | 1,72 | | 1,71 | 2,37 | 1,34 | 1,22 | 2,03 | 2,51 | 3,61 | 2,931 | - |
| #5 | 2,50 | 2,74 | 2,74 | 2,23 | 1,58 | 2,33 | 1,01 | 1,09 | | 1,65 | 1,32 | 0,74 | 0,89 | 1,46 | 1,10 | 2,66 | 1,661 | - |
| #6 | 0,13 | 0,20 | 0,20 | 0,18 | 0,16 | 0,13 | 0,09 | 0,10 | | 0,07 | 0,11 | 0,11 | 0,09 | 0,10 | 1,61 | 2,04 | 2,902 | - |
| #7 | **3,31** | **5,32** | **5,32** | **4,50** | **5,27** | **4,31** | **1,55** | **2,26** | | **1,99** | **2,59** | **1,89** | **2,26** | **2,13** | **1,61** | **2,54** | **2,675** | **Confirmed** |
| #8 | **4,18** | **6,74** | **6,74** | **4,75** | **5,13** | **5,03** | **1,85** | **2,54** | | **2,16** | **2,40** | **2,28** | **2,77** | **2,42** | **1,61** | **2,71** | **3,123** | **Confirmed** |
| #9 | 0,30 | 0,41 | 0,41 | 0,40 | 0,33 | 0,35 | 0,22 | 0,25 | | 0,23 | 0,28 | 0,25 | 0,24 | 0,24 | 1,38 | 1,68 | 1,776 | - |
| #10 | **0,00** | **0,01** | **0,01** | **0,01** | **0,01** | **0,01** | **0,00** | **0,01** | | **0,003** | **0,01** | **0,01** | **0,01** | **0,01** | **2,34** | **2,05** | **3,856** | **Confirmed** |
| #11 | 0,30 | 0,57 | 0,57 | 0,47 | 0,47 | 0,47 | 0,21 | 0,27 | | 0,21 | 0,28 | 0,27 | 0,25 | 0,25 | 1,88 | 2,21 | 2,717 | - |
| #12 | 0,48 | 0,61 | 0,61 | 0,56 | 0,59 | 0,54 | 0,32 | 0,37 | | 0,33 | 0,36 | 0,38 | 0,37 | 0,37 | 1,26 | 1,62 | 1,835 | - |
| #13 | **0,21** | **0,54** | **0,54** | **0,43** | **0,51** | **0,42** | **0,11** | **0,21** | | **0,06** | **0,22** | **0,20** | **0,20** | **0,19** | **2,50** | **2,72** | 8,940 | **Confirmed** |
| #14 | 0,56 | 0,77 | 0,79 | 0,59 | 0,60 | 0,71 | 0,38 | 0,42 | | 0,38 | 0,48 | 0,40 | 0,38 | 0,43 | 1,37 | 1,96 | 2,021 | - |
| #15 | **2,16** | **3,97** | **3,97** | **3,57** | **2,23** | **2,79** | **1,57** | **1,94** | | **2,25** | **2,30** | **1,86** | **1,65** | **1,91** | **1,83** | **2,20** | **1,763** | **Confirmed** |
| #16 | **0,14** | **0,21** | **0,21** | **0,20** | **0,17** | **0,17** | **0,10** | **0,11** | | **0,08** | **0,11** | **0,10** | **0,11** | **0,11** | **1,51** | **1,98** | **2,659** | **Confirmed** |
| #17 | **2,96** | **4,96** | **5,11** | **3,16** | **4,30** | **4,34** | **2,08** | **2,33** | | **2,6** | **2,54** | **2,01** | **2,21** | **2,51** | **1,68** | **2,28** | **1,909** | **Confirmed** |
| #18 | 0,32 | 0,40 | 0,40 | 0,40 | 0,34 | 0,34 | 0,24 | 0,26 | | 0,22 | 0,26 | 0,26 | 0,24 | 0,26 | 1,23 | 1,55 | 1,796 | - |
| #19 | 3,08 | 2,89 | 2,89 | 2,46 | 2,79 | 2,46 | 2,06 | 1,87 | | 2,13 | 1,94 | 1,76 | 2,01 | 1,71 | 0,94 | 1,58 | 1,357 | - |
| #20 | **2,73** | **4,11** | **4,11** | **4,04** | **3,76** | **4,10** | **1,76** | **2,49** | | **1,92** | **2,69** | **2,55** | **2,08** | **2,42** | **1,51** | **1,75** | **2,139** | **Confirmed** |

Table 2 shows the quantitative metrics obtained for the image database. SUV_max_ and SUV_mean_ indices are split into the different valvular regions (center; ring; descending aorta blood pool, BP; S1; S2; S3; S4). As mentioned, anatomical disparities lead to dissimilar orientation of the computed valvular segments. Valvular heterogeneity ratios VHI and RCR are obtained. Based on literature, the descending aorta blood pool ratio is computed.


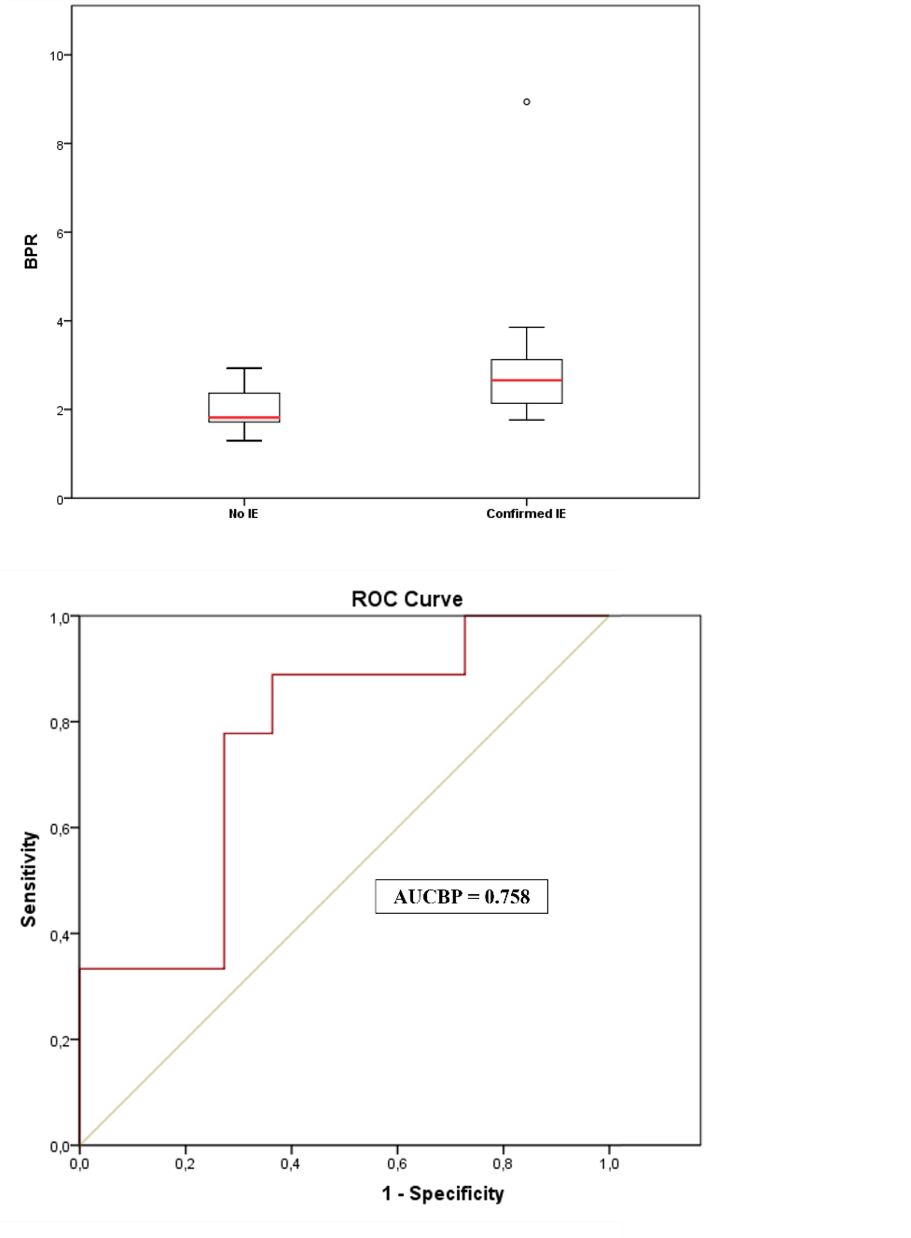


**Figure S1.** Boxplot and ROC curve for descending aorta blood pool ratio (BP).

**Table S2.** Descriptive statistics for the separated groups along the main valvular regions. Statistics for the uptake metrics and ratios are calculated. Statistically significant *p-*Value coefficients are given in **bold**.

| Metric | No IE | Confirmed IE | *p*-Value (Mann-Whitney) |
| --- | --- | --- | --- |
| SUV_max_ center | 1.26 ± 1.09 | 1.96 ± 1.52 | 0.47 |
| SUV_max_ ring | 1.70 ± 1.54 | 3.20 ± 2.45 | 0.21 |
| SUV_mean_ center | 0.80 ± 0.70 | 1.16 ± 0.84 | 0.34 |
| SUV_mean_ ring | 0.86 ± 0.70 | 1.50 ± 1.08 | 0.24 |
| VHI | 2.03 ± 0.63 | 2.24 ± 0.35 | 0.09 |
| RCR | **1.43 ± 0.44** | **1.79 ± 0.38** | **0.02** |
| BP ratio | 2,01 ± 0.58 | 3.25 ± 2.23 | 0.06 |
